# Surgical treatment features of liver gunshot wound with a dumdum bullet (expanding bullet)

**DOI:** 10.1186/s12245-022-00460-2

**Published:** 2022-10-10

**Authors:** Igor Lurin Anatoliyovych, Olexander Usenko Yuriyovych, Oleksandr Hrynenko Valentynovych

**Affiliations:** 1 National Academy of Medical Science of Ukraine, Kyiv, Ukraine; 2 National Institute of Surgery and Transplantation named after O.O. Shalimov NAMS of Ukraine, Kyiv, Ukraine; 3 Department of Liver Transplantation and Surgery, Shalimov National Institute of Surgery and Transplantation, 30 Heroiv Sevastopolya Str., Kyiv, 03680 Ukraine

**Keywords:** Liver injury, Abdominal injury, Abdominal trauma, Thoracoabdominal dumdum’s bullet trauma, Gunshot wound, Dumdum bullet (expanding bullet)

## Abstract

**Introduction:**

Liver injury is one of the most common abdominal traumas. The causes of military activity related injuries are gunshot wounds (up to 60–70%), while in peacetime—closed blunt abdominal trauma (up to 45–55%). The overall mortality is up to 40–60% and has higher rates in the group of wartime injury, among the male population over 65 years old and of low social status.

**Presentation of the case:**

We report the management of a clinical case of a 34-year-old man with thoracoabdominal dumdum’s bullet trauma in the case of which damage control tactics were applied in cooperation between two clinics in conditions of active hostilities.

**Discussions:**

Treatment of patients with abdominal injuries should be guided by the principles of damage control. This tactic requires stabilization of the patient's condition at the initial stage, followed by the completion of the final volume of surgical treatment in a compensated state of the patient. Liver injuries represent an ideal model for the application of damage control surgery in wartime settings and require close coordination between clinics that perform primary and delayed surgical interventions. Minimizing the volume of surgical intervention at the stage of primary control of bleeding due to liver damage provides the most optimal immediate results in conditions of a hemodynamically unstable patients, simultaneous admission of a large number of wounded, and a limited clinic resource.

**Conclusion:**

This surgical history research is an example of the effectively organized and coordinated work of two clinics such as National Military Medical Clinical Center “Main Military Clinical Hospital” and Shalimov National Institute of Surgery and Transplantation, based on the principle of damage control in conditions of active hostilities.

## Introduction

The term “expanding bullets” or so called “dumdum” refers to bullets that have a tendency to expand or flatten in the human body. This causes the bullet to increase in diameter, to combat over-penetration and produce a larger wound, thus dealing more damage to a living target. That is why, such type of bullets is prohibited for use during war time. A wide range of modern rifle and handgun bullets share some construction characteristics with the dumdum bullet, are intended to expand or flatten easily in the human body, and/or produce effects within the human body that resemble those of the dumdum bullet. Unfortunately, bullets that deform or mushroom immediately upon entering a body transfer the kinetic energy of the bullet to the tissue of the target more quickly. Such bullets are less likely to ricochet or to exit or penetrate an object or a person and wound other persons in the vicinity of the target (over-penetration), or to cause damage to surrounding objects. Due to these factors, and because in some cases they are claimed to have greater accuracy over long distances (useful for sniping), dumdum bullets are considered especially suited for use in situations where the target is in close vicinity of bystanders, respectively civilians, such as in an urban environment or other populated area.

Liver damage is one of the most common injuries in the abdominal cavity. Gunshot wounds are the cause of liver damage in up to 60–70% of cases in wartime conditions, while closed blunt abdominal trauma is up to 45–55% of cases in peacetime conditions [1]. In most cases, liver injuries are accompanied by simultaneous damage to adjacent organs (small intestine, large intestine, stomach, spleen). Twenty or 30 years ago, abdominal cavity gunshot wounds were absolute indications for emergency laparotomy. However, the development of damage control in the context of improving diagnostic methods and non-surgical control of bleeding has led to an increase in the number of patients with non-operative management. Most of liver injuries are minor or moderate (I–III grades according to World Society of Emergency Surgery and American Association for the Surgery of Trauma-Organ Injury Scale classifications, Table 1 and Table 2 respectively) and must be treated nonoperatively [2].

**Table 1:** World society of emergency surgery trauma-organ injury scale classification

	WSES grade	Blunt/penetrating (stab/guns)	AAST	Hemodynamic
Minor	WSES grade I	B/P SW/GSW	I-II	Stable
Moderate	WSES grade II	B/P SW/GSW	III	Stable
Severe	WSES grade III	B/P SW/GSW	IV-V	Stable
	WSES grade IV	B/P SW/GSW	I-VI	Unstable

*SW* stab wound, *GSW* gun shot wound, *AAST* American Association for the Surgery of Trauma

**Table 2:** American association for the surgery of trauma-organ injury scale classification

Grade	Injury type	Injury description
I	Hematoma	Subcapsular < 10% surface
	Laceration	Capsular tear < 1 cm parenchymal depth
II	Hematoma	Subcapsular 10–50% surface area; intraprenchymal, < 10 cm diameter
	Laceration	1–3 cm parenchymal depth, < 10 cm in length
III	Hematoma	Subcapsular > 50% surface area or expanding, ruptured subcapsular or parenchymal hematoma. Intraprenchymal hematoma > 10 cm
	Laceration	> 3 cm parenchymal depth
IV	Laceration	Parenchymal disruption 25–75% of hepatic lobe
	Vascular	Juxtavenous hepatic injuries, i.e., retrohepatic vena cava/centrl major hepatic veins
VI	Vascular	Hepatic avulsion

At the same time, severe liver damage combined with damage to GI organs and/or hemodynamic instability of the patient at the time of presentation is an absolute indication for laparotomy even in highly specialized centers. Based on the above tactics of managing a patient with liver damage should be based on the hemodynamic status of the patient, the presence of combined injuries requiring surgical intervention, but not an anatomical description of traumatic liver injury. Despite the improvement of both non-operative and operative methods of achieving hemostasis in liver injuries, mortality remains quite high and, in some cases, reaches 40–70%, being higher in the group of wartime injuries, among the male population over 65 years old and low social status.

## Presentation of case

**Fig. 1:**
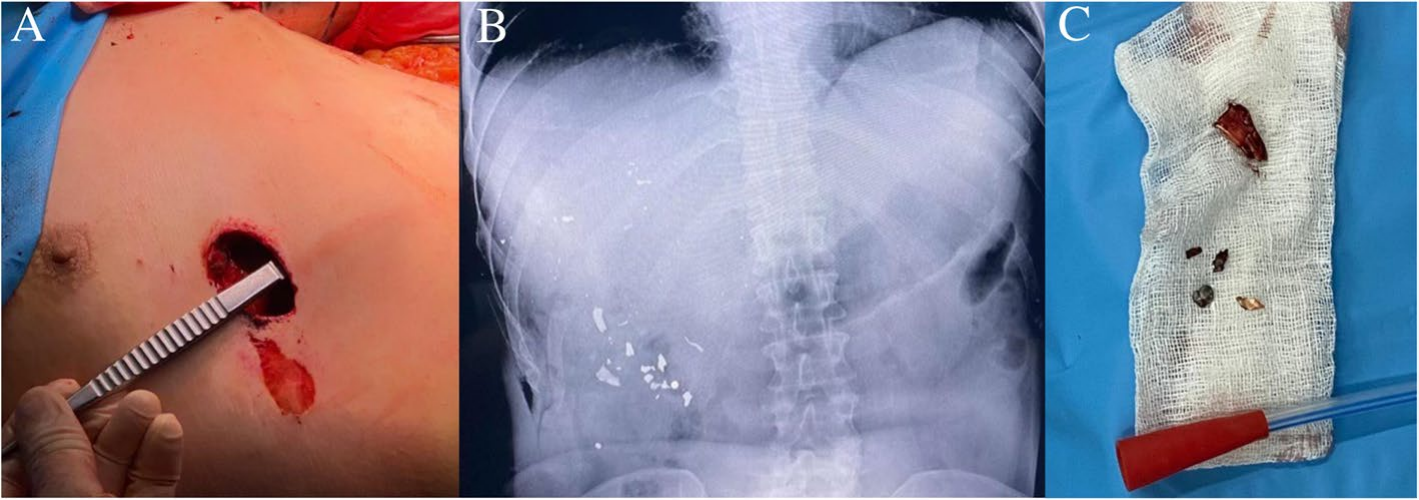
Nature of the primary destruction of the liver and fragments of the bullet

**Fig. 2:**
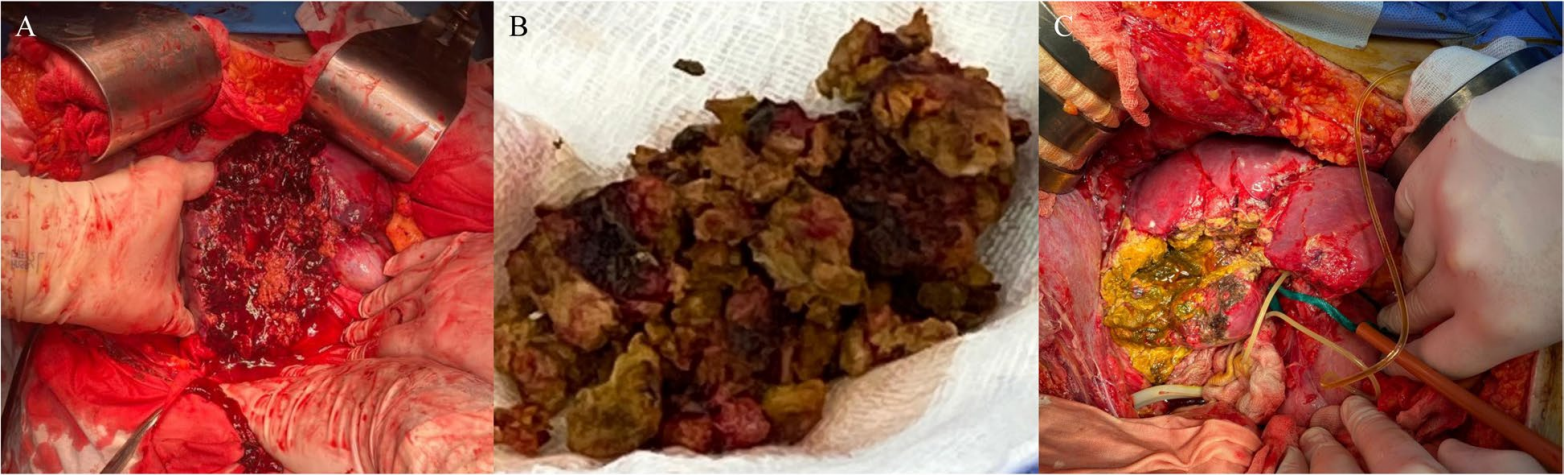
Intraoperative photo at initial liver operation

**Fig. 3:**
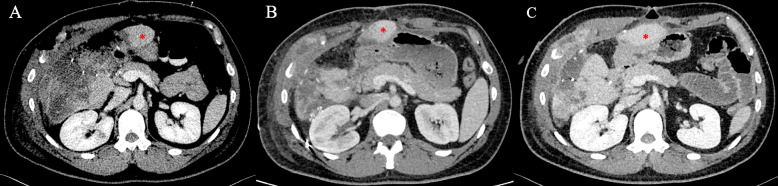
CT-scans that demonstrate improvement in perfusion in damaged segments of the liver

A 34-year-old man was transferred to the National Military Medical Clinical Center “Main Military Clinical Hospital” from the battlefield in an unconscious state with a gunshot wound in the right costal arch. At the time of admission, the patient received infusion therapy with crystalloid solutions with achievement of a systolic blood pressure of near 80 mmHg. Considering the hemodynamic instability of the patient, the presence of free fluid in the abdominal cavity revealed during FAST ultrasound, it was decided to perform an urgent surgical intervention according to the AAST protocols. Intraoperatively, a penetrating wound of the right dome of the diaphragm with an inlet in the projection of the right costal arch, rupture of the liver along the middle hepatic vein to the liver hilum, partial crush of 5**–**6 liver segments with massive hemoperitoneum, injury of the lower pole of the right kidney, fragment fractures of the ribs were revealed. With the highest degree of probability, an expanding bullet (dumdum) was used, as indicated by the large entrance hole, the nature of the primary destruction of the liver, and the fragments of the bullet that were removed during the operation (Fig. 1). Packing of the liver and perihepatic space, suturing of the lower pole of the right kidney, suturing of the diaphragm, external drainage of the common bile duct, and drainage of the subhepatic space with intraoperative stabilization of the patient’s condition were performed (Fig. 2A, B). On the postop day 1, bile leakage was detected through the drainage from the subhepatic space with a daily flow rate of up to 400 ml. On the postop day 2, the patient began to be disturbed by hyperthermia, despite this, his blood tests and general clinical condition were without features. On the postop day 3 due to the stabilization of the patient’s condition on the one hand and the need for further surgical intervention as part of damage control strategy, he was transferred to the Shalimov National Institute of Surgery and Transplantation. CT of the abdominal organs was performed at the time of admission and revealed a massive interlobar rupture of the liver along the median hepatic vein with parenchymal disruption of near 30% of the right lobe (Fig. 3). Performed cholangiography through the external drainage of the common bile duct showed the preservation of the bile duct confluence even with preservation of right anterior and right posterior bile ducts confluence. During reoperation, the previously installed gauze swabs were removed and a partial removal of necrotic areas of the liver parenchyma was performed (Fig. 2C). The areas of bile duct damage in the depths of the parenchymal disruption revealed by the bubble-test through the external drainage of the common bile duct were sutured with Maxon 5-0 and Maxon 6-0. Drainage of the right pleural cavity was performed due to massive pleural effusion. On the postop day 7, bile leakage into the wound of the right costal arch was detected. In this regard, US-guided percutaneous drainage of the biloma in the area of liver rupture was performed. During the next week, regular washings of the drainage were performed with the release of up to 40-50 ml of turbid bile daily. Repeated abdominal CT showed satisfactory perfusion of the damaged right lobe of the liver, which did not require any surgical interventions.

## Discussion

The tactics of managing patients with injuries of the parenchymal organs of the abdominal cavity has undergone significant changes over the past few decades. The principle of minimizing the primary operation, the purpose of which is, first of all, the stabilization of a critically ill patient, has replaced primary reconstructive-resection surgical interventions.

**Fig. 4:**
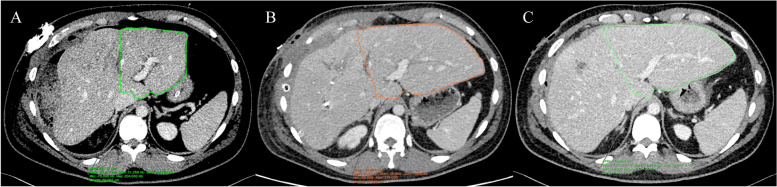
CT-scans that demonstrate regeneration of non-damaged left lobe of liver

The result of the application of damage control tactics was a significant improvement in the immediate and long-term results of the treatment of the wounded during the last military operations of our time [3]. Most often primary and repeated operations are carried out in different clinics. This in turn requires a clear understanding of the role of each treatment unit in a complex care chain based on damage control principles. Liver injuries are an ideal model for the use of injury control tactics, especially massive gunshot wounds in the context of a large simultaneous admission of patients to one clinic. Massive liver injury requires (WSES grade III**–**IV), first of all, adequate tamponade of the damage and the perihepatic space in the complex with the maximum allowable volume of ICU care—replenishment of blood loss and stabilization of the patient's hemodynamics. The degree of liver damage, in particular, depends on the type of the damaging element, in our clinical case we indicate a high probability of using an expanding bullet, as evidenced by a ballistic assessment of the injury of the wounded. Planimetric studies of expansive shells indicate that the increase in the wound canal is due to fragmentation of the wounding projectile and the cause of significant collateral plastic deformation of perivullar tissues, which leads to significant radial ruptures. Thus, the increase in the trauma of the expansive projectile is explained by the formation of a zone of secondary necrosis and molecular shock of tissues, which are the result of the existence of a temporary pulsating cavity, which is formed when the projectile enters the tissue [4]. The use of the latter in wartime conditions is prohibited by the principles of international humanitarian law [5]. Packing of the abdominal cavity carries the risk of infectious complications, therefore, careful monitoring of the patient's condition is aimed at choosing the optimal time for reoperation. In case of massive liver damage, a second operation is performed to remove the previously installed gauze swabs and assess the need for reconstructive or resection stages. The reconstructive stage concerns the elimination of damage to the bile ducts, which is most often presented in the form of targeted suturing of the source of bile leakage. On the other hand, the resection stage is a sparing removal of the necrotizing liver parenchyma. Even in spite of compensatory hypertrophy of the intact liver lobe (Fig. 4), major liver resections should be avoided due to the high risk of postoperative complications and mortality.

## Conclusion

Well-coordinated work of two independent medical institutions shows good immediate results in the application of damage control tactics for massive liver injury received during hostilities using extreme firepower weapons by the enemy. Dedicated to the heroic deeds of Ukrainian medical doctors in the name of life.

## Data Availability

All data generated or analyzed during this study are included in this published article.

## Abbreviations

WSES: World Society of Emergency Surgery
AAST: American Association for the Surgery Trauma
GI: Gastrointestinal (tract)
CT: Computed tomography scan
ICU: Intensive care unit
